# Evidence of Oropouche Orthobunyavirus Infection, Colombia, 2017

**DOI:** 10.3201/eid2706.204405

**Published:** 2021-06

**Authors:** Doris E. Gómez-Camargo, Jorge A. Egurrola-Pedraza, Cristopher D. Cruz, Dina Popuche, Margarita M. Ochoa-Díaz, Carolina Guevara, Maria Silva, Eugenio J. Abente, Julia S. Ampuero

**Affiliations:** Universidad de Cartagena, Cartagena, Colombia (D.E. Gómez-Camargo, J.A. Egurrola-Pedraza, M.M. Ochoa-Díaz);; US Naval Medical Research Unit No. 6, Lima, Peru (C.D. Cruz, D. Popuche, C. Guevara, M. Silva, E.J. Abente, J.S. Ampuero)

**Keywords:** Oropouche orthobunyavirus, *Bunyaviridae*, arboviruses, Colombia, Oropouche fever, viruses, vector-borne infections

## Abstract

We describe an Oropouche orthobunyavirus infection in a women 28 years of age in Colombia. We confirmed the diagnosis by viral isolation, quantitative reverse transcription PCR, and phylogenetic analysis of the small, medium, and large genomic segments. The virus is related to a strain isolated in Ecuador in 2016.

Oropouche fever is an emerging zoonotic disease caused by Oropouche orthobunyavirus (OROV; family *Peribunyaviridae*, genus *Orthobunyavirus*). The disease was initially reported in Trinidad and Tobago in 1955; since then, researchers have documented >30 outbreaks in Brazil and Peru and isolated cases in Panama and Ecuador (*1*,*2*). OROV infection is characterized by acute febrile illness with symptoms such as headache, myalgia, arthralgia, chills, photophobia, nausea, vomiting, and dizziness. Patients with severe cases might have hemorrhaging and aseptic meningitis (*1*). 

The OROV virion is enveloped and composed of a tripartite (segment lengths: 958 nt for small, 4,385 nt for medium, and 6,852 nt for large), negative-sense, single-stranded RNA genome (*1*,*3*,*4*). In 1964, Groot (*5*) described antibodies against OROV in serum samples from primates studied in Magdalena Medio and La Lizama (Colombia) in 1957. Since 2009, researchers have identified competent vectors such as *Aedes serratus*, *Coquillettidia venezuelensis*, and *Culex quinquefasciatus* mosquitoes on the Caribbean coast of Colombia (*6*,*7*). We describe an OROV infection in a woman in this region. We confirmed the diagnosis by viral isolation and reverse transcription PCR (RT-PCR).

A woman 28 years of age who did domestic work arrived at the emergency department of the E.S.E. Local Hospital of Turbaco (Turbaco, Colombia) on September 9, 2017. She had a 1-day history of fever, malaise, chills, myalgia, headache, retroocular pain, photophobia, dizziness, sore throat, anorexia, dysgeusia, and nausea. She had conjunctival injection and an axillary temperature of 38.6°C; she had no other pathologic abnormalities and tested negative on a tourniquet test. After receiving informed consent, we collected 12 mL of blood and stored the sample at −80°C.

One aliquot of serum was sent to the laboratory of the US Naval Medical Research Unit No. 6 (Lima, Peru) as part of an ongoing collaborative pathogen surveillance effort with the University of Cartagena (Cartagena, Colombia). This study protocol was approved by the Institutional Ethics Committee in Scientific Research of the University of Cartagena and the US Naval Medical Research Unit No. 6 Institutional Review Board (protocol no. NMRCD.2010.0010) in compliance with all applicable federal regulations governing the protection of human participants. 

We extracted RNA from the sample; it tested negative for dengue, Zika, and chikungunya viruses by real-time RT-PCR. We inoculated the sample into Vero 76 cells using a previously described technique (*8*) and observed a cytopathic effect in 50%–75% of the cells at 4 days after inoculation. We conducted an indirect immunofluorescence assay with pooled polyclonal antisera against flaviviruses (yellow fever virus and dengue virus serotype 3), alphaviruses (Venezuelan equine encephalitis virus, Eastern equine encephalitis virus, and Mayaro virus), hantavirus (Sin Nombre virus), arenaviruses (Allpahuayo virus and Tacaribe virus), cardiovirus (encephalomyocarditis virus), and bunyaviruses (Guaroa virus [GROV], caraparu virus, and OROV); we detected a positive signal with the bunyavirus antisera pool. We conducted another indirect immunofluorescence assay with individual polyclonal antisera against GROV, caraparu virus, and OROV; we detected a positive signal with the OROV polyclonal antisera. The serum sample tested negative for IgM against OROV, GROV, Mayaro virus, and Tacaribe virus by ELISA with whole virus antigen produced in-house. The original clinical samples tested positive by OROV-specific RT-PCR at independent laboratories in Peru and Colombia (*9*).

To characterize the virus at the molecular level, we used supernatant from the viral isolation to extract, amplify, and sequence the viral genome. We amplified the complete genome of the virus using a modified protocol of sequence-independent single primer amplification (*10*). We used the Nextera XT DNA Library Preparation Kit (Illumina, https://www.illumina.com) to prepare a library and sequenced the samples with MiSeq Reagent kit version 3 (600-cycle) (Illumina) according to the manufacturer’s instructions.

The phylogenetic analyses of the small, medium, and large segments (GenBank accession nos. MK643115–7) showed that the strain is closely related to a strain isolated in Ecuador in 2016; it also forms a clade with a strain isolated in Peru in 2008 (Figure). We cannot posit transmission dynamics across country borders without additional sequence data. The patient did not travel outside the municipality during the 15 days before symptom onset, suggesting that OROV transmission occurred in Turbaco. *Aedes serratus* and *Culex quinquefasciatus* mosquitoes, which are OROV-competent vectors, have been identified in Turbaco (E. Cano-Perez, pers. comm.). 

**Figure:**
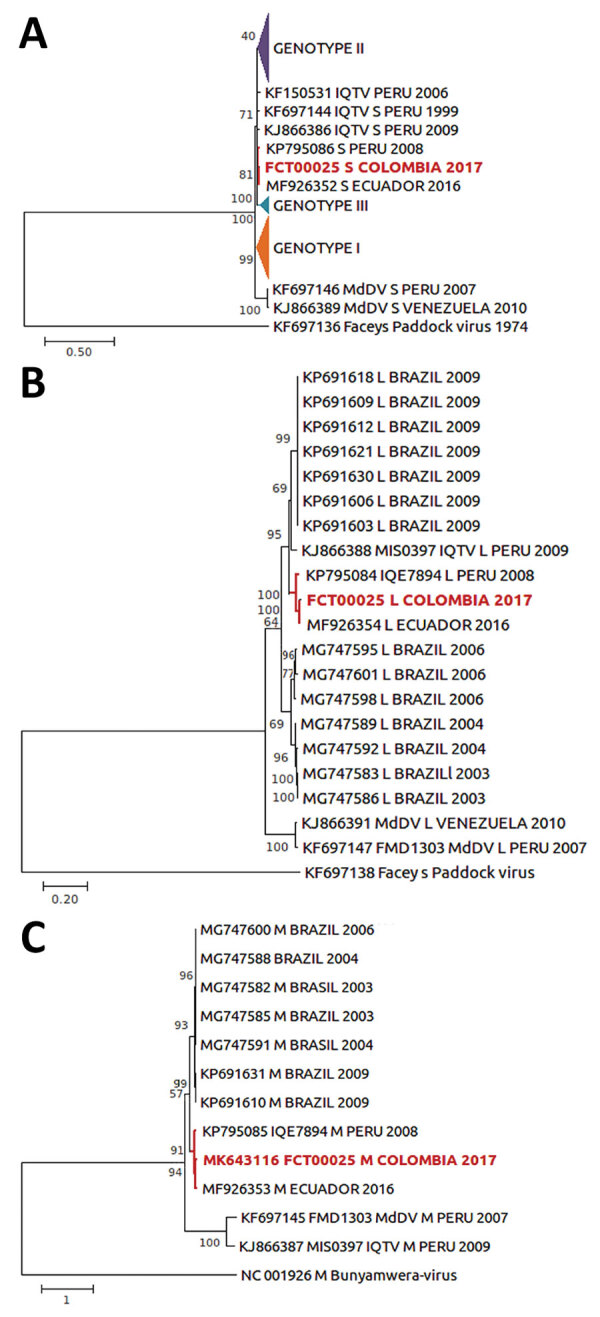
Maximum-likelihood phylogenetic tree based on the small (A), large (B), and medium (C) segments of Oropouche orthobunyavirus from a patient in Colombia, 2017 (red text), and reference sequences. Numbers to the left of nodes indicate bootstrap values based on 1,000 replicates. GenBank accession numbers are given for representative strains used for comparison. Triangles indicate phylogenetic branches compressed for size (*4*,*9*). Faceys Paddock and Bunyamwera viruses were used as outgroups. Scale corresponds to phylogenetic distance units estimated by likelihood function model. IQTV, Iquitos virus; MdDV, Madre de Dios virus.

Our findings support the diagnostic use of OROV-specific RT-PCR for patients with acute febrile illness in this region. The increased use of this diagnostic tool will help clarify levels of circulation of OROV, informing decisions about Force Health Protection of US service members. In conclusion, local transmission of Oropouche orthobunyavirus infection may be occurring in this region.
